# Buildings Contemplated and in Progress

**Published:** 1913-11-08

**Authors:** 


### Buildings Contemplated and in Progress.

BRADFORD.—The Local Government Board will shortly hold an inquiry into an application of the Corporation for authority to borrow £2,500 for the erection of a temporary smallpox hospital on a part of Odsal Tip-Lands.

BARNWOOD.—A committee has been appointed by the Gloucestershire County Council to consider proposals for a new male epileptic block and general bathing room for male patients at the Barnwood County Asylum.

COOTEHILL.—The Cavan and Monaghan Asylum Board are considering the question of converting the Cootehill Workhouse into an auxiliary mental hospital. In a report upon a recent inquiry into the matter the Local Government Board have indicated their willingness to accede to this proposal, provided the Cavan Board of Guardians spend £1,000 in making their workhouse suitable for patients.

DURHAM.—The Guardians have decided to purchase land on the north side of Red Hills Lane as a site for a workhouse infirmary.

LINCOLN.—As a result of representations from the Insurance Commissioners, who are urging the County Councils of Lincolnshire and the Borough of Grimsby to build a joint sanatorium, the Guardians of Lincoln have resolved to exclude the proposed phthisical block from the new infirmary about to be erected, and the beds will thus be reduced from 200 to 134.

LLANFOIST.—The Abergavenny Guardians have been authorised by the Local Government Board to borrow £305 for the purchase of a site for a new infirmary.

REDDISH.—The Stockport Town Council has resolved to purchase the Woodville estate at a cost of £6,500 for a sanatorium.

SEDGEFIELD.—The Guardians have been authorised to borrow £1,143 for a new infirmary block at the workhouse.

SHIRLEY.—The Corporation of Shirley are inviting tenders for constructing additions to the administrative block of the isolation hospital at Shirley. Specifications and particulars may be obtained from the Borough Engineer.

STOKE-ON-TRENT.—Plans for a children's hospital and nurses' home have been forwarded to the Local Government Board for approval. The estimated cost is £5,578.

